# Effects of “fresh mechanically deboned meat” inclusion on nutritional value, palatability, shelf-life microbiological risk and digestibility in dry dog food

**DOI:** 10.1371/journal.pone.0250351

**Published:** 2021-04-22

**Authors:** Giorgia Meineri, Alessia Candellone, Sonia Tassone, Pier Giorgio Peiretti, Erica Longato, Daniele Pattono, Natalia Russo, Elena Pagani, Liviana Prola

**Affiliations:** 1 Department of Veterinary Sciences, University of Turin, Grugliasco, Italy; 2 Department of Agriculture, Forestry, and Food Sciences, University of Turin, Grugliasco, Italy; 3 Institute of Sciences of Food Production, National Research Council, Grugliasco, Italy; 4 Faculty of Veterinary Science, Campus Aurelio Saliceti, University of Teramo, Teramo, Italy; 5 Monge & C. S.p.a., Monasterolo di Savigliano, Italy; University of Illinois, UNITED STATES

## Abstract

Fresh mechanically deboned meat (MDM) is usually claimed as high-quality ingredient on dry pet food recipes and this aspect may positively influence consumer choice. It is important to determine the scientifically sustainability of this claim and to assess the microbiological safety of MDM inclusion in dry pet food. Objectives were: 1) to evaluate the effect of inclusion of MDM in dry dog food on fatty acid profile and *in vivo* and *in vitro* digestibility, proposing a new system (Daisy^II^ Incubator) to measure the *in vitro* digestibility for dogs; 2) to compare palatability of dry dog food containing MDM with dry dog food in which meat by-products (MBP) are the only animal protein sources; 3) to determine, whether or not, the inclusion of that ingredient changes the microbiology and the storage quality. Results indicated that MDM product was characterized by significant higher nutritional value in terms of fatty acids profile, in vitro digestibility (HV-IVD method) and lower palatability than the MBP product. Microbiological risk assessment showed no microbiological hazards for either product. After 6-months storage, the total mesophilic bacterial count ranged between 1.77 and 2.09 log CFU/g feed, while polyamine values were higher in the MDM (0.37 g/kg) than in the MBP (0.27 g/kg). The Daisy^II^ Incubator was found to be a valid instrument for studying *in vitro* digestibility also for dogs, providing data simply, quickly, with less variability and costs than *in vivo* trials. In conclusion, MDM inclusion in dry dog food is microbiologically safe and it can improve its nutritional quality, at the expense of a reduced palatability. The higher polyamine levels fount in MDM-enriched petfood after 6-months storage, however, may represent a possible hazard, and further studies are still warranted.

## 1. Introduction

The majority of pet owners consider their pets to be family members (63% of pet owners in the United States and more than 71% in Italy), [1, 2]. Humanization of dogs and cats has directed the owners’ preferences for pet food claimed with “human‐grade” ingredients, mainly characterized by the replacement of processed animal proteins with raw animal protein sources (“fresh mechanically deboned meat”, MDM), and usually listed as the first ingredient on pet food labels. This trend in mainly driven by the erroneous belief that these products are safer, more palatable, more digestible and respond to human regulation [1]. Despite the “human-grade” claim have no definition in any animal feed regulations, and there aren’t supporting evidences of MDM inclusion on nutritional traits so far, the pet food industry is keen to increasingly include fresh and unprocessed chicken meat in dry pet food products.

In parallel, an unjustified demonization of the use of meat by-products in pet food has aroused [1].

Consumer uncertainty about what passes as “by-products” may lead pet owners to perceive them as poor-quality ingredients [3]. Processed animal proteins are, in fact, widely used in the pet food industry and provide an excellent source of protein, energy, and minerals [4, 5], however, misperceptions about their origin and content make fresh meat the more desirable ingredient.

Animal by-products are defined in article 3 of Regulation (EC) 1069/2009 as “entire bodies or parts of animals, products of animal origin or other products obtained from animals that are not intended for human consumption” [6]. Animal by-products can fall into three categories and category 3 material includes, among others, processed animal proteins usually purchased by manufacturers for pet food production. In details, “processed animal protein” means meat and bone meal, meat meal, bone meal, blood meal, dried plasma and other blood products, hydrolyzed protein, hoof meal, horn meal, poultry offal meal, feather meal, dry greaves, fishmeal, dicalcium phosphate, gelatin and any other similar products including mixtures, feeding stuffs, feed additives and premixtures, containing these products.

Fresh mechanically deboned meat, on the other hand, refers to meat that has not undergone any treatment except maintaining the cold chain. Treatments such as cooking, drying, freezing, hydrolysis or addition of preservatives exclude the component from being called “fresh” [7]. It is obtained by forcing pureed or ground pork, turkey or chicken meat, under high pressure through a sieve or similar device, to separate the bone from the edible meat tissue. These characteristics, together with the fact that MDM is usually listed as the first ingredient on pet food labels (due to the high- water content), could influence consumer choice among pet food products.

Including meat by-products or MDM in pet food can also have nutritional and technological implications. For example, rendering conditions, as well as thermal treatment and the source and handling of raw materials, can greatly influence nutritional and microbiological traits and digestibility of the protein meals [4, 8].

Most dry foods are produced by extrusion. Correct extrusion conditions favor higher retention of amino acids, high protein and starch digestibility, less lipid oxidation, and higher retention of vitamins [9]. In addition, the extrusion process denatures undesirable enzymes such as anti-nutritional factors (trypsin inhibitors, hemagglutinins, tannins, and phytates) and sterilizes the finished product [9–12]. Although overcooking can diminish the nutritional quality of foods, the relatively high moisture content, moderate temperatures, and short cooking duration all help to maintain the nutritional quality of extruded foods [9–12].

As regards microbiological issues, there have been several recalls of commercial pet foods and treats in the United States because of contamination with *Salmonella* spp., *Escherichia coli*, and other foodborne pathogens [13]. Contamination not only poses the risk that pets ingesting these food items can become clinically ill or may become carriers of the pathogens but it also represents a public health concern for pet owners who handle food products and interact with their pets [14, 15].

Finally, the use of animals in research elicits a diverse range of attitudes and emotions, with some people demanding the abolition of research on animals and others expressing strong support. Typically, opponents of animal research cite besides animal welfare and suffering also the uselessness of digestibility and palatability trials [16].

Given the above, the trend is to prefer the use of *in vitro* enzymatic analysis [17] over *in vivo* studies, which are more costly, laborious, and require animals anyway. In farm animal nutritional studies, *in vitro* digestibility is largely estimated using a Daisy^II^ Incubator (Ankom Technology Co., Fairport, NY, USA); this closed-system fermentation apparatus has been previously used in digestibility studies in ruminants [18, 19] and monogastric animals [20, 21]. Initially developed for multiple analysis of feeds, the incubator reduces labor demands, improves precision, and could offer an alternative system to traditional *in vitro* methods for pet food digestibility studies.

There are few studies to date that evaluate the effect of raw or fresh ingredients on pet food nutritional profile, conservation quality, palatability, and digestibility. The objectives of the present study were: (a) to evaluate the effect of inclusion of MDM in dry dog food on fatty acid profile and *in vivo* and *in vitro* digestibility, proposing a new system (Daisy^II^ Incubator) to measure the *in vitro* digestibility for dogs; (b) to compare palatability of dry dog food containing MDM with dry dog food in which meat by-products (MBP) are the only animal protein sources; (c) to determine whether or not there were differences in MDM/MBP pet food microbiology and conservation quality.

## 2. Materials and methods

This article does not contain any studies involving animals performed by any of the authors.

### 2.1. Diet formulation and extrusion parameters

Two isoenergetic and isonitrogenous extruded diets were formulated according to the National Research Council (NRC) guidelines for adult dogs [22], and prepared by a manufacturer located in Italy and authorized for pet food production according to EU legislation. One diet was formulated using mechanically separated chicken meat (MDM) as the first ingredient, while the other diet was formulated with processed animal protein (MBP) as the first ingredient.

Tables 1 and 2 present the mean chemical composition, inorganic mineral, vitamin, and amino acid content of the two feed materials and of the two diets, respectively. Nutrients determination was performed according to AOAC INTERNATIONAL standards (2018) [23].

**Table 1:** Chemical composition (g/kg dry matter), mineral content (g/kg dry matter), vitamin content and amino acid content (g/100 g dry matter) of the two types of feed materials (FM).

Items	MDM	MBP
DM (g/kg FM)	328	951
Crude protein	360	683
Ether extract	364	129
Nitrogen free extract	11	2
Cellulose	30	12
Ash	235	174
Ca	71	49
P	40	27
Mg	1.9	1.9
Zn	0.09	0.11
Fe	0.03	0.13
Vitamin B_1_ (mg/kg)	0.70	1.2
Vitamin B_2_ (mg/100g)	n.d.^1^	0.59
Vitamin B_12_ (μg/kg)	n.d.	0.04
Aspartic acid	2.4	5.3
Threonine	1.0	2.5
Serine	1.0	2.7
Glutamic acid	3.8	8.7
Proline	3.0	4.0
Glycine	5.5	6.7
Alanine	3.1	4.5
Valine	1.4	3.0
Methionine	0.24	1.3
Isoleucine	1.0	2.4
Leucine	1.8	4.4
Tyrosine	0.55	1.8
Phenylalanine	1.0	2.5
Lysine	1.6	4.3
Histidine	0.49	1.4
Arginine	2.6	5.1
Cysteine	0.12	0.43
Tryptophan	n.d.	0.33

n.d. = not detected.

**Table 2:** Chemical composition (g/kg, as-fed basis), mineral content (g/kg, as-fed basis), vitamin content and amino acid content (g/100 g, as-fed basis) of the two diets (MDM = Mechanically Deboned Meat diet and MBP = Meat By-Product diet; mean±SD).

Items	MDM^1^	MBP^2^
DM	940.5±0.5	930.1±0.3
Crude protein	263.1±0.2	264.7±1.0
Ether extract	176.8±0.3	187.0±2.7
Nitrogen free extract	401.4±0.8	373.8±4.8
Cellulose	26.6±0.9	24.1±0.6
Ash	72.6±0.9	80.6±1.5
Ca	15.6±0.8	18.4±4.7
P	10.7±0.4	12.4±3.0
Mg	1.41±0.07	1.41±0.34
Zn	0.28±0.01	0.27±0.01
Fe	0.29±0.01	0.26±0.03
Vitamin B_1_ (mg/kg)	12.1±0.1	10.1±0.2
Vitamin B_2_ (mg/100g)	1.47±0.01	1.28±0.02
Vitamin B_12_ (μg/kg)	0.11±0.01	0.09±0.00
Vitamin A (U.I/kg)	30267±896	31000±854
Vitamin D_3_ (U.I/kg)	2153±693	2280±324
Vitamin E (mg/kg)	194±17	189±7
Aspartic acid	2.30±0.03	2.29±0.03
Threonine	1.07±0.02	1.08±0.02
Serine	1.22±0.02	1.16±0.02
Glutamic acid	3.62±0.05	3.50±0.07
Proline	1.64±0.09	1.56±0.04
Glycine	2.32±0.04	2.45±0.05
Alanine	1.74±0.02	1.78±0.05
Valine	1.30±0.01	1.36±0.04
Methionine	0.67±0.02	0.54±0.02
Isoleucine	1.04±0.06	1.04±0.06
Leucine	2.11±0.01	1.99±0.03
Tyrosine	0.82±0.03	0.66±0.07
Phenylalanine	1.14±0.02	1.15±0.01
Lysine	1.50±0.06	1.60±0.04
Histidine	0.56±0.02	0.56±0.01
Arginine	1.80±0.03	1.84±0.04
Cysteine	0.26±0.01	0.24±0.02
Tryptophan	0.15±0.01	0.13±0.00
Metabolisable Energy (kJ/Kg)	16789	16998

^1^MDM ingredients: mechanically separated chicken meat, processed animal protein, rice, potato protein concentrate, animal fat, maize, beet pulp, brewer’s yeast, hydrolyzed animal protein, *Spirulina platensis*, *Yucca schidigera*, hydrolyzed cartilage, hydrolyzed crustaceans, methyl sulfonyl methane, *Echinacea* root, oregano, garlic, vitamin and mineral mix.

^2^MBP ingredients: processed animal protein, rice, potato protein concentrate, animal fat, maize, beet pulp, brewer’s yeast, hydrolyzed animal protein, *Spirulina platensis*, *Yucca schidigera*, hydrolyzed cartilage, hydrolyzed crustaceans, methyl sulfonyl methane, *Echinacea* root, oregano, garlic, vitamin and mineral mix.

Specific extrusion parameters used by the manufacturer during petfood production are reported in Table 3.

**Table 3:** Production of MDM (Mechanically Deboned Meat) diet and MBP (Meat By-Products) diet and relative processing temperature, modified by [24–26].

Phase	Duration (min)	MDM Temperature (°C)	MBP Temperature (°C)
Preparation of the main ingredient mixture	-	Room temperature	Room temperature
Pre-grinding storage	-	Room temperature	Room temperature
Grinding	60	40°	40°
Grinding mixture storage	180	Room temperature	Room temperature
Pre-conditioning	1	From 25° to 60°	From 25° to 60°
Extrusion	0.5	From 85° to 110°	From 70° to 110°
Drying	30	From 85° to 160°	From 50° to 95°

### 2.2. Fatty acid analysis

To determine fatty acid composition of the diets, lipid extraction and gas chromatography (GC) were performed according to Peiretti and Meineri [27]. All analyses were performed in triplicate.

### 2.3. Microbiological analyses and shelf-life

Microbiological analysis was conducted to determine the microbiological quality of ingredients, intermediate feed, throughout the production stages (where possible contamination may occur), and feed at the end of production and at 6 months of storage under controlled conditions in samples from a box of the same batch. All microbiological methods were ISO [28] or taken from the literature: ISO 6579:2002 for Salmonella also referred to pet food and ISO 4833-1991/AFNOR V08-054 for the total mesophilic bacterial count. All media were Oxoid (Basingtoke, UK), except for Chromocult® (Merck, Germany). Microbial counts are expressed as a logarithm (log) of colony-forming units (cfu) per gram of sample. Quantitative analysis was carried out in duplicate.

Briefly the methods:

- Total mesophilic bacterial count: plate count agar at 30°C for 72 h;
- Enterobacteriaceae and *E*. *coli* counts: violet bile glucose agar and Chromocult® (Merck, Germany), respectively. Plates were incubated at 37° C for 24–48 h;
- Salmonella count: 50 g were suspended in buffered peptone water for pre-enrichment at 37°C for 18 h, Rappaport-Vassiliadis medium with soya (RVS broth) at 41.5°C for 24 h and Muller-Kauffmann tetrathionate/novobiocin broth (MKTTn broth) at 37°C for 24 h for selective enrichment, then plated the third day on xylose lysine dextrose agar (XLD) and brilliant green agar (BGA) incubated at 37°C for 24 h; phenotipical identification was carried out on Kligler iron agar and genotypical identification was performed according to Rahn et al. [29];
- sulfide reductase clostridium: SPS medium at 37° for 48 h under strict anaerobic conditions [30].

Biogenic amines were determined according to Paulsen et al. [31] on a high-performance liquid chromatography visible UV detector (HPLC UV/Vis). Peroxide determination was performed according to the EU official method [32]. Analyses were performed in triplicate.

### 2.4. Palatability trial

To evaluate the palatability of MDM and MBP, the two-bowl trial method was used [33]. This trial was prospectively approved by Ethical committee of the University of Turin. Briefly: MDM and MBP were placed in their respective bowls and presented simultaneously to a panel that judged food palatability according to three sensory characteristics: aroma, texture, and macronutrient profile. The panel was composed of 40 adult dogs of any breed and size randomly divided by sex and individually caged at feeding time; for the remainder of the day they were housed in kennels in groups of 20. The trial lasted 24 h during which each dog was presented once, for 30 minutes, with the two bowls and had to choose from which to eat first. The bowls contained 500 g of MDM or MBP. The quantities of each product consumed (intake) and left (outtake) were recorded for each dog. At the end of the trial, the quantities of MDM and MBP consumed by the whole panel were summed to determine which of the two diets was consumed more. Data are expressed as the total amount of food consumed for each diet, intake ratio (IR), preference, and food selected as a first choice. Water was supplied *ad libitum*.

### 2.5. In vivo digestibility trial

The digestibility protocol followed the guidelines published in the official method of the Association of American Feed Control Officials [34]. The trial was prospectively approved by Ethical committee of the University of Turin. The *in vivo* digestibility (Vd) was tested in 10 Beagles (5 males and 5 females) weighing between 8 and 16 kg. All dogs had been part of a trained panel. The animals were individually housed during the trial. In order to test the digestibility of MDM and MBP, the same dogs were employed for both trials but with different schedules according to the official guidelines [6]. Each Vd trial consisted of 3 days of adjustment to the new diet, followed by a 4-day collection period during which the weight of food offered and refused, and feces were recorded daily. A total of 500 g/day was offered as fed, while water was supplied *ad libitum*. The food was presented in the morning for 30 min. The total individual daily feces were weighed and then kept at -18°C pending analysis. The feces samples of each dog were dried; the 4-day cumulative samples were pooled from the daily samples. Apparent digestibility is expressed as g/kg. The total tract apparent digestibility coefficients of DM were calculated for each diet according to Villaverde et al. [35] using the formula:

Vd(g/kg)=[(Xintakeg‐Xouttakeg)/Xintakeg]x1000 (1)

Housing and treatment protocols (Registration Number 612.623.82) adhered to European norms for animal welfare [34, 36]. The facility was maintained according to regulations [37].

### 2.6. In vitro digestibility trials

*In vitro* digestibility was estimated using a Daisy^II^ Incubator (Ankom Technology, Macedon, NY, USA). The incubator has four digestion jars which rotate at constant and uniform temperature inside a temperature-controlled chamber. Each jar is filled with an enzymatic solution and can hold up to 23 filter bags with samples and one blank.

Filter bags (F57, Ankom Technology Corp.) were pre-rinsed in pure acetone (99%) in order to remove surfactants, which might block the bag pores and inhibit the enzyme activity. Bags were air-dried and numbered using a solvent resistant marker.

The extruded diets, oven-dried and grounded through a 1 mm screen, were weighed (0.5 ± 0.01 g) in triplicate into the filter bags. The heat-sealed bags were put in the jars with the enzyme and buffer solution, and then digested. Two digestibility techniques, differing in phosphate buffer, type and amount of enzymes were used to simulate the digestion. The first solution (HV-IVD), as proposed by Hervera et al. [38], and the second (BG-IVD), as proposed by Biagi et al. [39] were prepared and used as described in Table 4. The enzymes were pepsin (P7125, Sigma Aldrich), pancreatin (P1500, Sigma Aldrich), and bile salt. The phosphate buffer of the HV-IVD solution contained the acid (KH_2_PO_4_) and its conjugate base (K_2_HPO_4_), [40]. For the BG-IVD solution, the phosphate buffer was prepared as described by Martillotti et al. [23]. During the digestion time, the jars were slightly agitated and kept at constant temperature (39°C). At the end of incubation, the bags were removed from jars and rinsed thoroughly with warm tap water (39°C) with little agitation. Samples were rinsed until the water was clear. Before oven dry over-night at 65–70°C, the sample bags of the HV-IVD method were rinsed in ethanol and acetone by complete immersion for about 5 minutes, respectively. Later, bags were weighted and the final weight was recorded to calculate *in vitro* digestibility (IVD) as follows:

$$IVD\left(g/kg\right)=\left[\left(DM_{ante\;incubation}-DM_{post\;incubation}\right)/DM_{ante\;incubation}\right]\;x\;1000$$ (2)

**Table 4:** *In vitro* digestibility using the Daisy^II^ Incubator and according to the method described in Hervera et al. [38], (HV-IVD) and in Biagi et al. [39], (BG-IVD).

Step	HV-IVD	BG-IVD
	0.5 ± 0.01 g of sample^1^	0.5 ± 0.01 g of sample
1 Gastric digestion	1200 ml phosphate buffer (0.1M, pH 6) 480 ml HCl (0.2M) 480 mg pepsin 24 ml cloramphenicol solution (0.5g/100 ml ethanol) pH 2, 39°C, 2 h	1440 ml of pepsin-lipase-HCl solution (HCl 0.075N; pepsin 2g/L; gastric lipase 1g/L) 39°C, 2 h
2 Post-gastric digestion	480 ml phosphate buffer (0.2 M, pH 6.8) 240 ml NaOH (0.6 M) 4.8 g pancreatin pH 6.8, 39°C, 4 h	1440 ml -pancreatin-bile salt-phosphate buffer solution (10g/L pancreatin 25 g/L; bile salt) pH 7.5, 39°C, 4 h
3 Collection of undigested fraction	F57 washed, twiced with ethanol (96%) and twice with acetone (99%), dried overnight at 70°C, analysed for CP, EE, DM, OM	F57 washed, dried overnight at 65°C, analysed for CP, EE, DM, OM

^1^ Quantities for each jar and twenty-four F57 filter bags (twenty-three replicate samples and one blank).

### 2.7. Statistical analysis

Fatty acid content and data from the digestibility and palatability trials were analyzed using SPSS software (version 11.5.1 for Windows, SPSS Inc., USA) by one-way ANOVA with diet as the main factor; storage quality parameters were analyzed by ANOVA for multifactorial analysis of variance for the two main factors (diet and conservation time) to identify differences. Time effect (month 0 and 6), diet effect (MDM vs. MBP) and the time×diet interaction were considered to be statistically significant at P <0.05.

## 3. Results

### 3.1. Fatty acid profile

Table 5 presents the fatty acid composition of the two diets. MDM was richer than MBP in polyunsaturated fatty acids (PUFAs), whereas MBP was higher in saturated fatty acids (SFAs) than MDM, because MDM had higher values for linoleic acid and lower values for some SFAs (capric, lauric, myristic, and palmitic acid). MDM also had higher values for other SFAs (pentadecanoic and margaric acid) and MUFAs (heptadecanoic and elaicid acid).

**Table 5:** Fatty acid profile (mean±SD; g/100 g of total fatty acid) of the two diets (MDM = Mechanically Deboned Meat; MBP = Meat By-Products) and the significance between them.

Compound	MDM	MBP	*P*
Capric acid (C10:0)	n.d.^1^	0.02±0.03	-
Lauric acid (C12:0)	0.12±0.01	0.42±0.01	0.000
Myristic acid (C14:0)	1.16±0.02	1.21±0.01	0.025
Myristoleic acid (C14:1)	0.22±0.03	0.23±0.01	0.692
Pentadecanoic acid (C15:0)	0.16±0.01	0.15±0.00	0.016
Palmitic acid (C16:0)	20.69±0.03	20.78±0.03	0.020
Palmitoleic acid (C16:1n7)	4.85±0.03	4.80±0.03	0.123
Margaric acid (C17:0)	0.27±0.01	0.24±0.00	0.001
Heptadecanoic acid (C17:1)	0.24±0.01	0.22±0.01	0.013
Stearic acid (C18:0)	6.24±0.10	6.12±0.04	0.108
Oleic acid (C18:1n9)	40.64±0.15	40.58±0.11	0.592
Elaidic acid (C18:1n9, trans)	0.54±0.02	0.41±0.01	0.000
Linoleic acid (C18:2, cis-cis)	22.60±0.14	22.23±0.02	0.012
Linoelaidic acid (C18:2, trans-trans)	0.13±0.03	0.11±0.10	0.706
α-Linolenic acid (C18:3n3)	1.96±0.02	1.33±1.15	0.403
Arachidic acid (C20:0)	0.15±0.01	0.15±0.01	1.000
Behenic acid (C22:0)	n.d.	0.02±0.03	-
Eicosenoic acid (C20:1)	0.49±0.04	0.61±0.10	0.374
Lignoceric acid C24:0)	0.13±0.02	0.14±0.01	0.275
SFA^2^	28.91±0.09	29.23±0.02	0.004
MUFA^3^	46.45±0.10	46.44±0.02	0.878
PUFA^4^	24.55±0.16	24.25±0.05	0.036

^1^ n.d. = not detected.

^2^SFA = saturated fatty acid.

^3^MUFA = monounsaturated fatty acid.

^4^PUFA = polyunsaturated fatty acid.

### 3.2. Microbiological profile and shelf-life

Microbiology results are shown in Table 6 (ingredients) and Table 7 (after extrusion, after drying, and final feed formula at time 0 and after 6 months of storage under controlled conditions). All ingredients had a variable and high microbiological count for mesophilic bacteria and Enterobacteriaceae (range from 1.48 log CFU/g to 5.73 log CFU/g). *Clostridium* spores and *E*. *coli* were not detected in any samples.

**Table 6:** Mean counts (mean±SD, log CFU/g) of the main microbial groups detected in the ingredients.

Ingredient	Total Mesophilic Bacterial Count	Enterobacteriaceae	*E*. *coli*	*Clostridium* Sulfite Reductase	Salmonella^1^
Chicken meat	5.28±2.45	3.20±1.84	1.95±0.62	n.d.^2^	n.d.
Meat by-products	5.23±2.32	2.97±1.75	n.d.	n.d.	Positive
Rice	5.73±2.49	3.11±1.84	n.d.	n.d.	n.d.
Potatoes	5.43±0.01	n.d.	n.d.	n.d.	n.d.
Chicken Fat	1.48±0.84	n.d.	n.d.	n.d.	n.d.
Maize	4.59±1.83	2.58±1.32	n.d.	n.d.	n.d.
Beet Pulp	4.20±1.70	2.51±1.19	n.d.	n.d.	n.d.
HAP^3^	3.15±2.05	3.70±1.35	n.d.	n.d.	n.d.
Spiruline	3.66±1.62	n.d.	n.d.	n.d.	n.d.

^1^ Determined on 50 g.

^2^ n.d. = not detected.

^3^ HAP = hydrolyzed animal protein.

**Table 7:** Mean counts (mean±SD log CFU/g) of the main microbial groups) in the two diets (MDM = Mechanically Deboned Chicken Meat diet and MBP = Meat By-Products diet) at different phases.

Diet	Phase^1^	Total Mesophilic Bacterial Count	Enterobacteriaceae	*E*. *coli*	*Clostridium* Sulfite Reductase	Salmonella^2^
MDM	1	2.08±1.19	n.d.^3^	n.d.	n.d.	n.d.
MDM	2	2.15±1.05	n.d.	n.d.	n.d.	n.d.
MDM	3	2.08±0.75	n.d.	n.d.	n.d.	n.d.
MDM	4	2.10±1.19	n.d.	n.d.	n.d.	n.d.
MBP	1	1.85±0.62	n.d.	n.d.	n.d.	n.d.
MBP	2	1.48±0.90	n.d.	n.d.	n.d.	n.d.
MBP	3	1.70±0.92	n.d.	n.d.	n.d.	n.d.
MBP	4	1.78±0.92	n.d.	n.d.	n.d.	n.d.

^1^ Phase 1: after extrusion, Phase 2: after drying, Phase 3: at start of conservation period, Phase 4: after 6 months of conservation.

^2^ Determined on 50 g.

^3^ n.d. = not detected.

*Salmonella* spp. was detected in meat by-product before extrusion, but never after extrusion. Biomolecular analysis confirmed the identification after culture of *Salmonella enterica*. After 6 months of storage under controlled conditions, the microbiological profile was confirmed: the total mesophilic bacterial count ranged between 1.77 log CFU/g and 2.09 log CFU/g feed. Enterobacteriaceae, *Clostidium*, and *E*. *coli* were under the detection level and Salmonella was never detected.

The microbiological risk assessment showed no microbiological hazards for the use of MDM or MBP.

Table 8 reports the polyamine content of the two types of meat, with lower content of putrescine, spermidine, and spermine in MDM than MBP, that had cadaverine, histamine, and tyramine. Table 9 shows that among the polyamines evaluated, only phenylethylamine, histamine and tyramine contents were greater in MDM than MBP; putrescine, phenylethylamine, histamine, tyramine, and peroxide value were significantly increased, while spermine was decreased after 6 months of storage in both diets.

**Table 8:** Natural polyamines (mean±SD, mg/kg, as-fed basis) of the two types of meat (fresh and meal).

Compound	Mechanically Deboned Meat	Meat By-Products
Putrescine	1.5±0.1	78.6±96.7
Cadaverine	n.d.^1^	164±214
Tryptamine	n.d.	n.d.
Phenylethylamine	n.d.	6.3±0.2
Spermidine	17.2±0.5	35.0±35.7
Spermine	32.7±7.5	60.1±75.5
Histamine	n.d.	14.1±24.5
Tyramine	n.d.	61.2±69.7

^1^ n.d. = not detected.

**Table 9:** Polyamine (mean±SD mg/kg, as-fed basis) and peroxide content (meq/kg, as-fed basis) of the two diets (MDM = Mechanically Deboned Chicken Meat diet and MBP = Meat By-Products diet) at the start and the end of the conservation period.

Diet	MDM		MBP		Diet	Time	Diet x Time
**Time (month)**	**0**	**6**	**0**	**6**	***P***	***P***	***P***
Putrescine	56.9±2.3	94.5±14.2	58.3±3.7	74.2±11.2	0.137	0.001	0.092
Cadaverine	88.4±3.9	112.9±17.1	89.8±6.3	87.4±13.3	0.187	0.306	0.139
Tryptamine	n.d.^1^	15.7±2.5	n.d.	n.d.	-	-	-
Phenylethylamine	4.3±0.5	7.1±1.3	3.9±1.0	4.2±1.0	0.032	0.047	0.088
Spermidine	18.1±2.5	19.6±3.0	25.4±2.8	18.6±2.9	0.055	0.171	0.055
Spermine	7.3±2.1	3.5±0.6	13.8±5.4	2.1±0.5	0.101	0.013	0.034
Histamine	10.3±0.6	28.5±4.4	7.7±0.3	16.7±2.6	0.002	0.001	0.126
Tyramine	52.9±2.6	93.7±14.3	36.5±3.6	67.3±10.3	0.003	0.001	0.310
Peroxide value	1.9±0.2	6.0±0.8	2.5±0.4	6.0±0.8	0.213	0.001	0.709

^1^ n.d. = not detected.

### 3.3. Palatability

**Fig 1:**
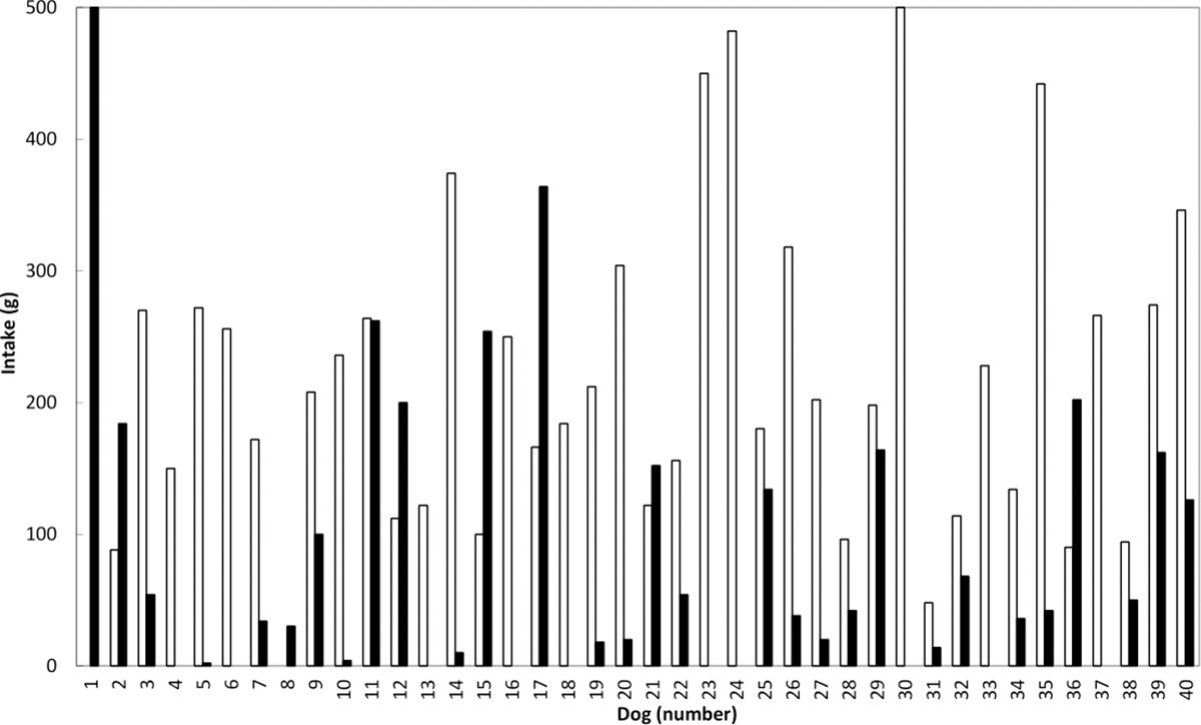
Summarized food intake of the two diets [MDM = Mechanically Deboned Chicken Meat (black columns) and MBP = Meat By-Products (white columns)] for each dog (*P*<0.001).

**Fig 2:**
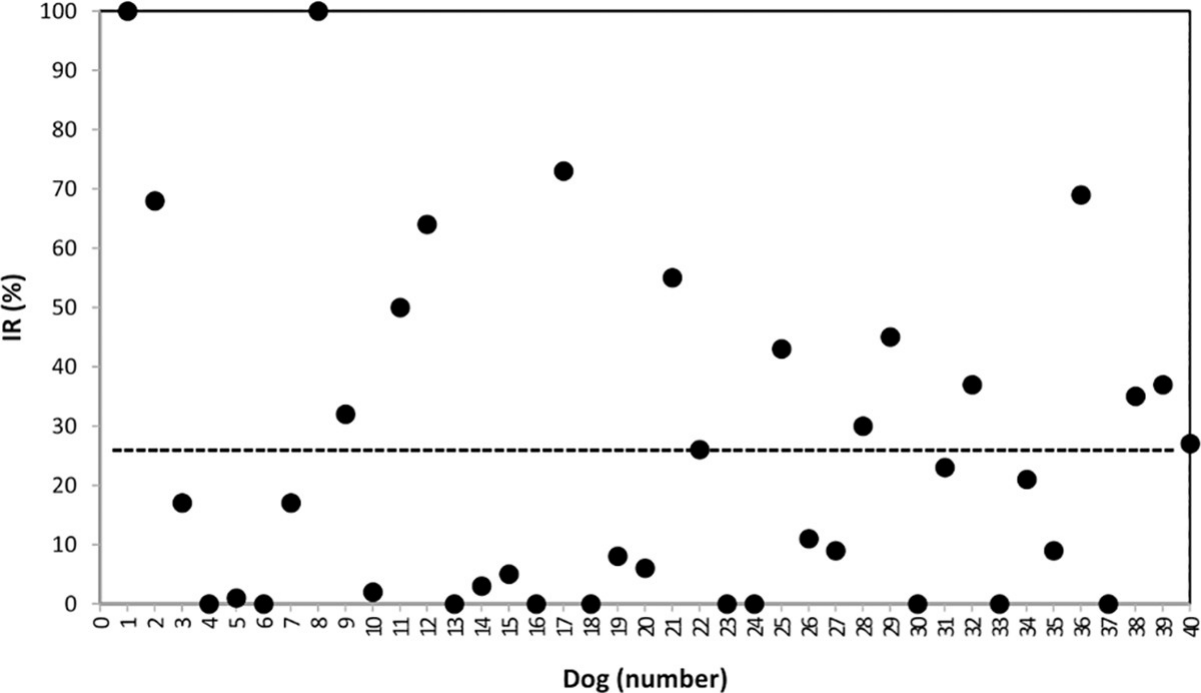
Intake Ratio [IR% = MDM/(MDM + MBP) x 100] of the MDM diet (based on Mechanically Deboned Chicken Meat) as compared with the MBP diet (based on Meat By-Products) for each dog (———Mean value).

From statistical point of view, the palatability trial showed higher (P<0.001) intake of MBP than MDM (Fig 1) with a mean intake value of 212.0 g vs. 83.5 g, respectively. The MDM diet alone was consumed by a single subject and was preferred by only 4 out of the 40 dogs with a percentage consumption of 27.1% and a percentage first choice of 33%. MBP was appreciated by all the remaining dogs and in 6 subjects exclusively, with a percentage consumption of 72.9% and a percentage first choice of 67%. To eliminate the difference related to breed and food intake, the Intake Ratio of the MDM diet was corrected for total intake per subject and a mean value of 25.6 was found (Fig 2).

### 3.4. *In vivo* and *in vitro* digestibility

**Fig 3:**
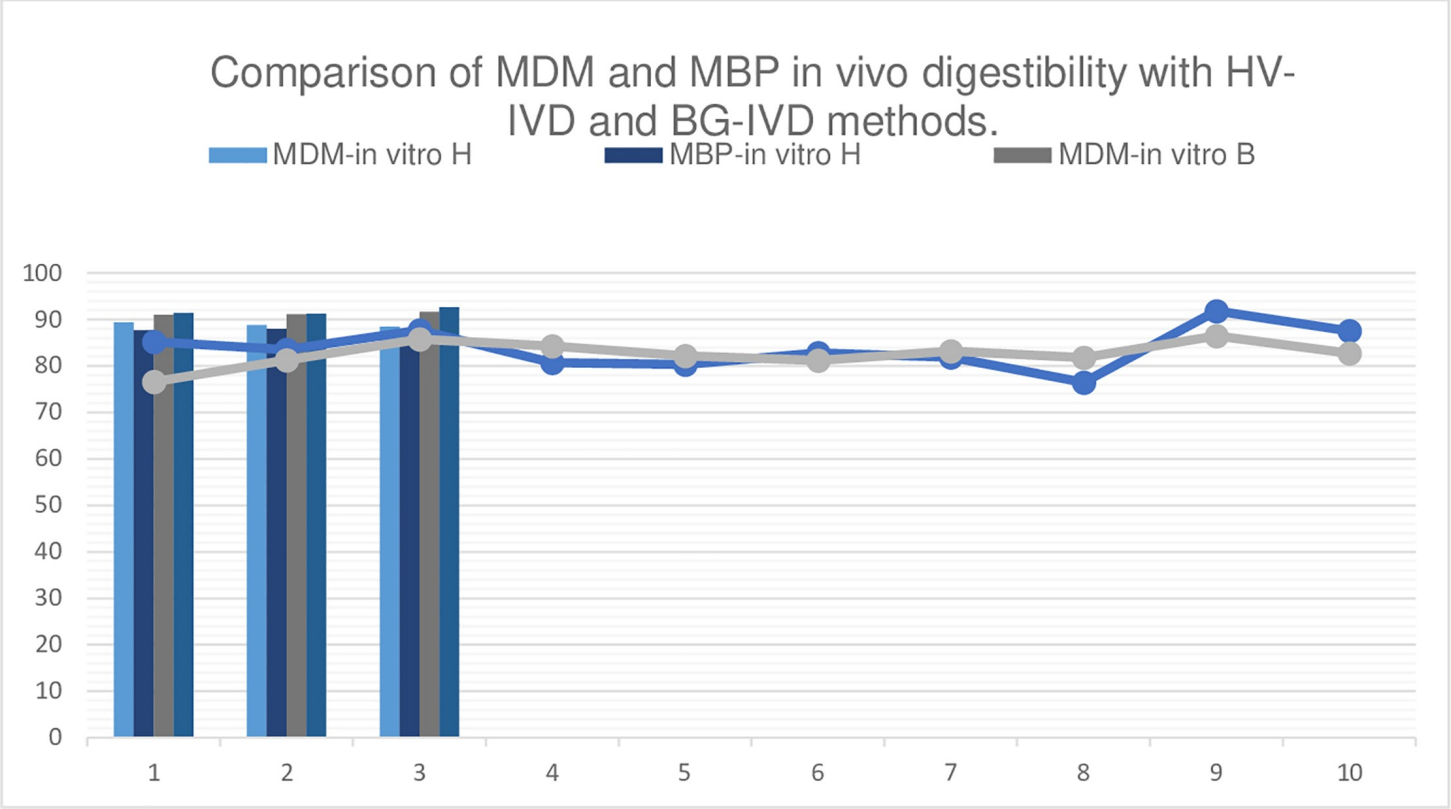
Comparison of MDM and MBP in vivo digestibility with HV-IVD and BG-IVD methods.

Table 10 presents the results of the *in vivo* and *in vitro* trials of diet digestibility. The *in vivo* method revealed no significant differences in digestibility between the two feeds. As measured by the *in vitro* method, HV-IVD revealed a significant difference between diets, whereas BG-IVD did not. It can be observed, however, that, as compared with the *in vivo* digestibility value, the *in vitro* method slightly overestimated the digestibility coefficients for both diets (Table 10 and Fig 3).

**Table 10:** *In vitro* dry matter digestibility (g/kg) according to the Hervera et al. method (HV-IVD), [38] and according to the Biagi et al. method (BG-IVD), [39] and *in vivo* dry matter digestibility (*in vivo*) of the two diets (MDM = Mechanically Deboned Chicken Meat diet and MBP = Meat By-Products diet) and the significance between them.

Digestibility method	MDM	MBP	*P*
*In vivo*	838.0±44.2	825.4±27.6	0.453
HV-IVD	888.8±0.5	877.9±0.1	0.021
BG-IVD	912.5±0.3	917.5±0.8	0.360

## 4. Discussion

### 4.1. Fatty acid profile

The MDM diet had better nutritional quality with higher content of PUFAs and lower content of SFAs than the MBP diet, with possible positive implications for some categories of animals (young and old dogs or animals with special needs such as hunting dogs). The PUFA n-6/PUFA n-3 ratio was 11.5 and 16.7 for the MDM and the MBP diet, respectively. These ratios are within the range reported by Kearns et al. [41], who studied the effect of diets formulated to contain PUFA n-6/PUFA n-3 ratios of 5 or 25 on oxidative status and immune response of young and old dogs. They found that a diet with a PUFA n-6/PUFA n-3 ratio of 5 had a positive effect on the immune response of young or geriatric dogs. Hall et al. [42] compared three diets with a PUFA n-6/PUFA n-3 ratio of 1.4, 18, and 40, respectively, fed to geriatric dogs and concluded that the dose of PUFA n-3 administered determined the plasma PUFA n-3 composition, independent of the PUFA n-6/PUFA n-3 ratio. This difference for fatty acid composition could be addressed to the preservation of fat naturally occurring in fresh meat while in MDM diet, the rendering process remove the natural fat and a fat with more standard composition is then added to the final product.

### 4.2. Microbiological profile and shelf-life

The microbiology of pet food has been associated with certain zoonoses [43–45]. Our results clearly show high microbiological counts for all the ingredients. If we compare our data with the few published data, this is not surprising given the origin of the food [46, 47]. Due to the combination of pressure and temperature, the extrusion process reduced the bacterial count by between 3 and 4 -log in both formulations. This result is in agreement with previous studies [30, 44, 45].

The total mesophilic bacterial count was lower than previously reported [30, 43]. While this parameter is relatively high for a final product with a long shelf-life, in our opinion and as stated by other authors the very low water activity (Aw of 0.40) can easily counteract bacterial growth [30]. The same behavior was noted for Enterobacteriaceae: their counts decreased from 3.70 log CFU/g to below the detection limit (1 log CFU/g) in both formulations. These results evidence a better situation for this batch than reported by other authors [29, 43]. Also, as seen for other dry pet food formulations, the *E*. *coli* count was consistently under the detection limit [30].

Salmonella, a highly common zoonotic pathogen responsible for causing infection in pets and owners [43–45], was detected only in untreated raw ingredients, in agreement with Van Bree et al. [47]. Sampling at three different time points in the production chain, where contamination could be expected, gave negative results. Our results show that, for this batch, extrusion treatment was safe, as reported elsewhere in the literature [30, 44, 45]

Furthermore, after 6 months storage there was a slight increase in microbiological parameters in both formulas, as observed by other authors [30]. Our results show that MDM appears more susceptible to degradation than MBP. Natural polyamines are organic compounds that originate from amino acids as a result of decarboxylation. This process can result from bacterial activity and can occur during food processing or storage. The most common monoamines, (histamine, tyramine, and tryptamine) and polyamines (putrescine and cadaverine) are generated, respectively, from the amino acids histidine, tyrosine, tryptophan, ornithine, and lysine; whereas, spermidine and spermine derive from putrescine. Polyamines are found in many protein foods and their amount is an important indicator of the degree of freshness and storage of products [48].

Studies on changes in polyamine content in meat products are inconsistent, however. Changes in polyamine content during meat storage result from bacterial activity. Ruiz-Capillas and Jiménez-Colmenero [49] reported that the polyamine level in minced meat products increased due to muscle fiber disintegration and increased microbial contamination. Putrescine levels in fresh meat are usually low, often near the limit of detection of the analytical procedures used [50]. The only amines present at significant levels in fresh meat are spermidine and spermine [50]. This is in agreement with the results obtained in our study (Table 8).

Regarding the storage quality of the product, after 6 months of storage, the polyamine levels were higher for MDM than MBP (Table 9). Polyamine synthesis requires the availability of amino acid precursors that may be present in the food product. As shown in Table 4, MDM contained more tyrosine and tryptophan than MBP, which are two of the amino acid precursors of tyramine and tryptamine, respectively. Some amines (e.g., tyramine, putrescine, and cadaverine) can form during the meat preservation [51]. Paulsen et al. [31] analyzed 55 samples of canned pet food and found that 75% of the levels of the biogenic amines varied between 5.4 and 21.9 mg/kg, depending on the type of amine, while it ranged from below the detection limit to 133 mg/kg for cadaverine, histamine, phenylethylamine, putrescine, and tyramine. They concluded that amine concentrations in non-fish components are lower than in fish components.

### 4.3. Palatability

The palatability trial showed a significant increase of MBP intake (72.9%) and difference in intake between the MBP and the MDM diet could have been due to several different factors. First, the composition of the product: the MBP diet was fatter (200 g/kg DM) than the MDM (188 g/kg DM). In fact, dogs prefer fat-rich diets [42] to high-protein or carbohydrate diets. Another factor is the drying process following extrusion (which reduced the humidity to 6% in MDM and to 7% in MBP); for MDM product, the minimum temperature was higher than that used for MBP (85°C vs. 50°C) due to the higher water content of the extruded product. Moreover, MDM diet showed higher polyamines content after 6 months of storage. This aspect, in literature, is described to be responsible for a palatability reduction [42, 52, 53].

### 4.4. Digestibility

The *in vitro* digestibility was high for both diets; results were higher than those reported by Brambillasca et al. [54] for two dry food measured *in vivo* (83.3% and 70.5%). This could be explained by the positive effect of extrusion that allows gelatinization of starches and increases digestibility in comparison with the pelleting process [55]. However, Biagi et al. [39] reported an average of 70.4% for the digestibility of extruded diets for dogs, which contrasts with the average reported in the literature (82.2%) and reported in their article.

Moreover, our data show different results obtained from the two methods and use of the Daisy^II^ Incubator. According to HV-IVD, digestibility was higher for the MDM diet than the MBP diet, (888.8 vs. 877.9 g/kg). Since the chemical composition of the MDM diet had less ether extract—though cellulose content was higher (26.6 vs. 24.1 g/kg), it contained higher quantities of starch (274.7 vs. 248.3 g/kg). Increasing the fiber level in dog diet decreases its digestibility [54], which is negatively related (r = - 0.86) to apparent digestibility of extruded food [55]. BG-IVD did not reveal significant differences between MDM and MBP (912.5 g/kg vs. 917.5 g/kg). *In vitro* values were always higher than *in vivo* digestibility and less variable, particularly with the BG-IVD method. The standard deviation ranged between 0.1 and 0.8 g/kg *in vitro* and 27.6 and 44.2 g/kg *in vivo*. Both *in vitro* methods used in this study were carried out in three steps using different types and amounts of enzyme: a higher amount of enzymes was used for BG-IVD than HV-IVD. Regmi et al. [56] found in pigs that *in vitro* digestibility was greater with longer digestion time, while the amount of enzymes was irrelevant for the digestibility.

Previous studies have shown that the ANKOM method produces digestibility values comparable to traditional procedures for many foods [57–62]. The *in vitro* method proposed by Hervera et al. [38] and utilized in our study yielded values closer to *in vivo* results, in line with Hervera et al. [63, 64] who showed the highest accuracy approach of *in vivo* crude protein apparent digestibility (r = 0.81) and *in vivo* digestible energy (r^2^ = 0.94), respectively.

## 5. Conclusions

Our results indicate that MDM and MBP are both a high-quality source of animal protein for commercial dry pet food. MDM diet demonstrated a higher nutritional value in terms of fatty acids profile (with higher content of PUFAs and lower content of SFAs) and in vitro digestibility (exclusively by HV-IVD method). Microbiological risk assessment revealed no microbiological hazards for the use of either MDM or MBP diet. After 6-months storage, the total mesophilic bacterial count ranged between 1.77 and 2.09 log CFU/g feed, while polyamine values were higher in the MDM (0.37 g/kg) than in the MBP (0.27 g/kg). Simultaneously, MDM diet demonstrated lower palatability compare to MBP diet, maybe related to the higher polyamine values. Furthermore, the Daisy^II^ Incubator was found to be a valid instrument for studying *in vitro* digestibility also for dogs, providing data simply, quickly, with less variability and costs than *in vivo* trials. It could represent the future for digestibility studies in pet food. In conclusion, MDM inclusion in dry dog food is microbiologically safe and it can improve its nutritional quality, at the expense of a reduced palatability. The higher polyamine concentrations found in MDM-enriched petfood after 6-months storage, however, may represent a possible risk. Indeed, threshold levels of biogenic amines in petfood have not been established so far, and further studies investigating their possible hazard in companion animals are still warranted.

## Abbreviations

MDM: fresh mechanically deboned meat
MBP: meat by-products
HV-IVD method: *in vitro* digestibility, as proposed by Hervera et al., (2007)
BG-IVD: *in vitro* digestibility, as proposed by Biagi et al. (2016)
